# Effectiveness and cost-effectiveness of an intervention to improve Initial Medication Adherence to treatments for cardiovascular diseases and diabetes in primary care: study protocol for a pragmatic cluster randomised controlled trial and economic model (the IMA-cRCT study)

**DOI:** 10.1186/s12875-022-01727-6

**Published:** 2022-07-05

**Authors:** Alba Sánchez-Viñas, Carmen Corral-Partearroyo, Montserrat Gil-Girbau, M. Teresa Peñarrubia-María, Carmen Gallardo-González, María-del-Carmen Olmos-Palenzuela, Ignacio Aznar-Lou, Antoni Serrano-Blanco, Maria Rubio-Valera

**Affiliations:** 1 Health Technology Assessment in Primary Care and Mental Health (PRISMA) Research Group, Institut de Recerca Sant Joan de Déu, Santa Rosa 39-57, 08950 Esplugues de Llobregat, Spain; 2 Centro de Investigación Biomédica en Red de Epidemiología y Salud Pública (CIBERESP), Madrid, Spain; 3 Facultat de Medicina i Ciències de la Salut, Universitat de Barcelona, c. Casanova 143, 08036 Barcelona, Spain; 4 Department of Paediatrics, Obstetrics, Gynaecology and Preventive Medicine, Univ Autonoma de Barcelona, Bellaterra, Spain; 5 Parc Sanitari Sant Joan de Déu, Doctor Antoni Pujadas 42, 08830 Sant Boi de Llobregat, Spain; 6 Centre d’Atenció Primària Bartomeu Fabrés Anglada, Direcció D’Atenció Primària Regió Metropolitana Sud, Institut Català de la Salut, Barcelona, Spain; 7 Unitat de Suport a la Recerca Regió Metropolitana Sud, Fundació Institut Universitari per a la recerca a l’Atenció Primària de Salut Jordi Gol i Gurina (IDIAPJGol), Barcelona, Spain

**Keywords:** Primary care, Complex intervention, Shared decision-making, Medication adherence, Cost-effectiveness analysis, Economic model, Cardiovascular disease

## Abstract

**Background:**

Between 2 and 43% of patients who receive a new prescription in PC do not initiate their treatments. Non-initiation is associated with poorer clinical outcomes, more sick leave and higher costs to the healthcare system. Existing evidence suggests that shared decision-making positively impacts medication initiation. The IMA-cRCT assesses the effectiveness of the IMA intervention in improving adherence and clinical parameters compared to usual care in patients with a new treatment for cardiovascular disease and diabetes prescribed in PC, and its cost-effectiveness, through a cRCT and economic modelling.

**Methods:**

The IMA intervention is a shared decision-making intervention based on the Theoretical Model of Non-initiation. A cRCT will be conducted in 24 PC teams in Catalonia (Spain), randomly assigned to the intervention group (1:1), and community pharmacies in the catchment areas of the intervention PC teams. Healthcare professionals in the intervention group will apply the intervention to all patients who receive a new prescription for cardiovascular disease or diabetes treatment (no other prescription from the same pharmacological group in the previous 6 months). All the study variables will be collected from real-world databases for the 12 months before and after receiving a new prescription. Effectiveness analyses will assess impact on initiation, secondary adherence, cardiovascular risk, clinical parameters and cardiovascular events. Cost-effectiveness analyses will be conducted as part of the cRCT from a healthcare and societal perspective in terms of extra cost per cardiovascular risk reduction and improved adherence; all analyses will be clustered. Economic models will be built to assess the long-term cost-effectiveness of the IMA intervention, in terms of extra cost for gains in QALY and life expectancy, using clinical trial data and data from previous studies.

**Discussion:**

The IMA-cRCT represents an innovative approach to the design and evaluation of behavioural interventions that use the principles of complex interventions, pragmatic trials and implementation research. This study will provide evidence on the IMA intervention and on a new methodology for developing and evaluating complex interventions. The results of the study will be disseminated among stakeholders to facilitate its transferability to clinical practice.

**Trial registration:**

ClinicalTrials.gov, NCT05026775. Registered 30^th^ August 2021.

**Supplementary Information:**

The online version contains supplementary material available at 10.1186/s12875-022-01727-6.

## Background

### Prevalence and impact of non-initiation

Medication adherence is a broadly studied health problem with a high impact on clinical outcomes and mortality [1–3]. Studies have mainly focused on persistence-related problems, such as early discontinuation, and implementation-related problems, like suboptimal dosing [4, 5]. Recently, a growing interest in adherence problems at the moment of initiating a medication has arisen [6–8]. Initiation is defined as the moment “when the patient takes the first dose of a prescribed medication” [4]; therefore, adherence problems related to initiation occur in cases of “late or non-initiation of the prescribed treatment” [4].

Recent studies indicate that up to 43% of new treatments are not initiated [7, 8] and that the prevalence of non-initiation is between 6 and 28% in Primary Care (PC) in the European context [9–11]. Non-initiation is associated with higher costs to the healthcare system, mostly generated by productivity losses and an increased number of home visits (which suggest worse disease progress) [12, 13], representing an economic burden for healthcare systems in the short term [12–14].

### Cardiovascular Disease and Diabetes

Cardiovascular disease (CVD) and diabetes are highly prevalent diseases with high morbimortality, they are the leading causes of death worldwide [15], and have a significant social and economic impact [16, 17].

Between 2 and 43% of treatments for CVD and diabetes are still not initiated [7, 11]. Early discontinuation and poor treatment implementation are also highly prevalent. For example, early discontinuation rates range between 11% (statins) and 18% (ECA inhibitors) [18], and more than 30% of patients who initiated treatment for CVD and/or diabetes abandon it within the first 3 years [19].

Studies on non-adherence to CVD and diabetes treatments found that it worsens the control of the disease [20–23] thus increasing morbidity, mortality [19, 24, 25] and healthcare costs [14]. Even though these studies have focused on persistence and implementation, it is expected that non-initiation may add to these negative effects [6].

### Effectiveness of strategies aimed to improve initiation

Different approaches have been used to address adherence [26, 27] and the evidence suggests that multi-component and theory-based interventions have the best chance of improving adherence [28, 29].

Systematic reviews identified a series of factors related to the disease, treatment, patient and the healthcare system that affect the probability of initiation, including the absence of social support, the cost of treatment, patients’ age and country of origin and beliefs about medication [7, 8, 30]. However, results from quantitative studies do not completely explain this phenomenon.

A few studies have explored the motivations for non-initiation to medications using qualitative methods [31–35]. The Theoretical Model of Medication Non-initiation [34, 35] shows that users make a risk–benefit assessment of new prescriptions which is influenced by their beliefs regarding the disease and the medication, their feelings, health literacy and other cultural factors, as well as the relationship between the patient and the Health System (especially the general practitioner [GP] and the pharmacist) and their context [34, 35]. Fear of adverse effects, doubts about the effectiveness of the medication, pill burden, preference for lifestyle interventions and cost of treatment also affect initiation [31–35].

Previously, not much effort had been made to address non-initiation. Only 9 randomised controlled trials (RCTs) have been conducted to assess the impact of interventions on non-initiation; none evaluated a theory-based intervention and they were conducted in the United States [36–44]. Three studies were conducted in secondary care [36–38]; those combined technical and educational interventions but did not have a positive impact on initiation. Among the six studies that were conducted in the PC context of the United States, some consisted of reminders for patients, which increased treatment purchases [39–42, 44]. However, adherence is heavily affected by desirability bias and false negatives are common when patients feel observed [45–47]. Consequently, it is likely that patients only purchased medication when they were aware that health professionals knew that they had not filled their prescriptions. The last study was also based on reminders and aimed to identify and resolve barriers to adherence but neither had an impact on non-initiation [43].

### The Initial Medication Adherence (IMA) study

The IMA study has an effectiveness-implementation hybrid design [48]; it consists of a pragmatic cluster randomised controlled trial (cRCT) along with a process evaluation to understand the effect of the IMA intervention in terms of effectiveness and cost-effectiveness, and to redefine the intervention before its implementation. Hybrid designs aim to evaluate the effectiveness of interventions while gathering information for their implementation in clinical care [49, 50] and are expected to speed the translation of research findings into routine practice [49, 50].

The IMA intervention was developed within the Medical Research Council (MRC) Framework for Complex Interventions [51, 52]. Further details on the design of the intervention and process evaluation are described elsewhere [53].

Following the MRC guidelines, several studies were carried out to identify the evidence base and develop the theory on which the IMA intervention is based. Using real-world data (RWD), the prevalence of non-initiation was estimated to be 17% in Catalan PC; for CVD and diabetes treatments specifically, it ranged between 5.7% (ACE inhibitors) and 9.1% (antiplatelet). Factors explaining non-initiation were also identified [9, 18, 30]. Additionally, to understand patients’ motivations for non-initiation, two qualitative studies based on Grounded Theory were conducted [34, 35]. The results of these studies were used to generate the Theoretical Model of Medication Non-Initiation [34, 35]. Finally, the evidence on interventions aiming to improve initiation was reviewed.

An initial version of the IMA intervention was drafted taking into account all the available evidence; it was based on the Theoretical Model of Medication Non-Initiation [34, 35]. It is a multidisciplinary intervention that promotes health literacy and shared-decision making (SDM) to improve medication initiation and secondary adherence and reduce cardiovascular risk (CVR).

To increase the acceptability and transferability of the intervention, discussion groups were then conducted with GPs, nurses, community pharmacists and other healthcare professionals, who made suggestions for optimisation, defined the limitations of the intervention and anticipated barriers to its implementation.

Before a definitive cRCT, a pilot study with an integrated process evaluation was conducted to assess the feasibility and acceptability of the IMA intervention and to test the clinical trial design [54], and the IMA intervention was optimised and refined accordingly to its results [53].

The aim of the present paper is to describe the study protocol for the cRCT of the IMA intervention.

## Methods/design

### Study aims

The aims of the Initial Medication Adherence–cluster-randomised controlled trial (IMA-cRCT) are, first, to assess the effectiveness of the IMA intervention compared to usual care in improving medication initiation, secondary adherence and clinical outcomes in patients who have been prescribed a new treatment for CVD or diabetes in PC using a cRCT; and second, to evaluate the cost-effectiveness of the IMA intervention, in comparison to usual care, in terms of extra cost per reduction of cardiovascular risk, gains in quality-adjusted life years (QALY) and life-years gained (LYG) using a cRCT and economic modelling.

### Design

**Fig. 1:**
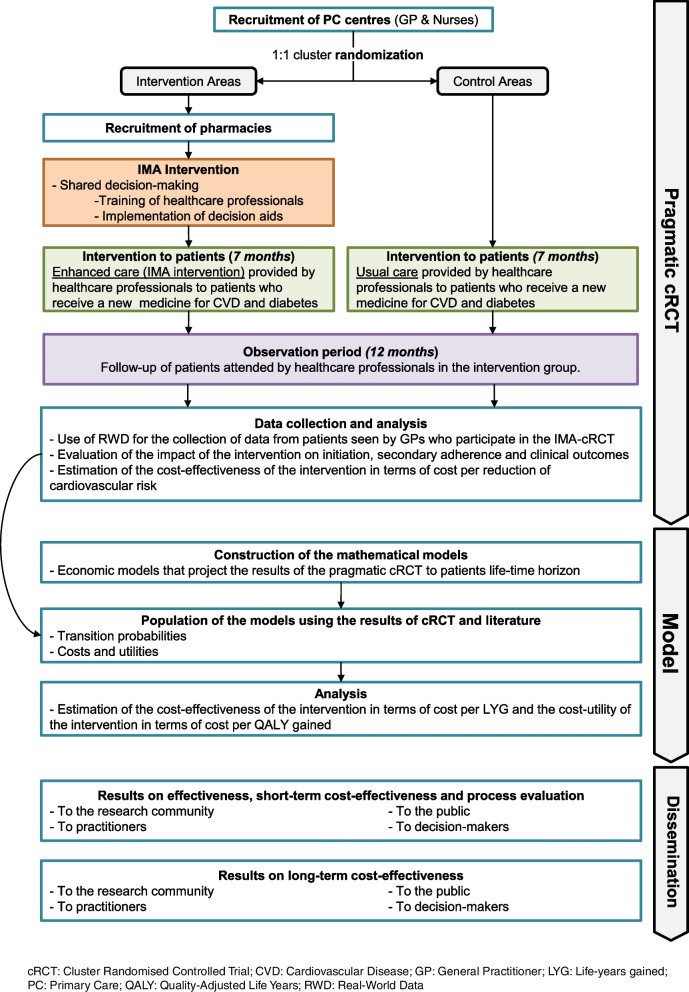
Summary of the IMA-cRCT Research Project and timeline

The IMA-cRCT study consists of a 7-month pragmatic cRCT with a 12-month follow-up, with an integrated process evaluation to understand the trial results and refine the intervention accordingly (methods are detailed elsewhere [53]), and economic modelling to provide long-term evidence of the cost-effectiveness and cost-utility of the IMA intervention. Figure 1 shows the summary of the IMA-cRCT study.

The intervention assignment is cluster-based considering PC teams and two parallel arms: usual care in the control group and the IMA intervention in the intervention group. Twenty-four PC teams will participate in the trial.

The Standard Protocol Items: Recommendations for Interventional Trials (SPIRIT) checklist for study protocols [55, 56] is provided in Additional file 1. Details about the pragmatic design of the IMA-cRCT are provided in the Pragmatic Explanatory Consortium Indicator Summary (PRECIS-2) [57] wheel scheme in Additional file 2.

### Setting

The study will be carried out in the Spanish PC setting. PC health centres and community pharmacies from urban and rural areas in Catalonia, a Spanish region of four provinces with a population of 7.6 million people [58], will be included in the study.

The Spanish National Health System offers universal coverage, is funded from taxes and health service provision is mostly within the public sector. Most competencies of this public system are transferred to the seventeen regions which manage and organise public healthcare services within their area [59]. In Catalonia, the Catalan Health Service (CatSalut) is responsible for managing and organising the services of the public healthcare system. CatSalut outsources the provision of services with not-for-profit private and public providers. Its main health service provider at the PC level is the Catalan Health Institute, a public provider, covering 80% of the population [60].

PC is the point of access to the public system, acting as a gatekeeper for secondary care. PC manages the highest volume of prescriptions, which are issued by GPs and dispensed by community pharmacists. Following Clinical Practice Guidelines, GPs carry out health promotion and prevention, early detection, treatment and monitoring of the most prevalent health problems. Nurses also perform health promotion and prevention actions; additionally, they provide direct patient care by assessing patients’ needs, planning and delivering adequate care and evaluating the results. In recent years, SDM is being promoted in the healthcare community, although this patient-centred approach is recommended but not yet standardised.

Medications under study can be exclusively obtained at pharmacies with a prescription but patients have freedom of choice on which community pharmacy to use to buy prescribed medicines. Although healthcare professionals are encouraged to work in an interdisciplinary way, due to the context of the Spanish National Health System, it is difficult for PC professionals to work in coordination with community pharmacists regarding the management of prescriptions or medication [61, 62]. In Spain, Community pharmacies are considered private health establishments of public interest [63]. Pharmacies can have more than one owner but at least one of the owners must be a pharmacist [64]; however, each pharmacist can only be the owner of one pharmacy. There is no limit on pharmacists or pharmacy technicians working in community pharmacies and although the former are responsible for dispensing medication, technicians can also dispense medication under the supervision of a pharmacist. For this reason, there must be at least one pharmacist working in the pharmacy at all times.

In 2019, the Council of Official Colleges of Pharmacists of Catalonia, in collaboration with CatSalut, developed an alert embedded in the e-prescription system to inform pharmacists when a patient is starting a new treatment. This is aimed to help pharmacists to provide adequate information while dispensing the new treatment [65]. This tool is available in all Catalan pharmacies for some medications, including platelet aggregation inhibitors excluding heparin, and insulins, regardless of the IMA intervention.

The Spanish National Health System provides free-of-charge services (outpatient and inpatient care) with some exceptions, such as outpatient pharmaceutical prescriptions, which are subjected to cost-sharing for patients. The contribution is based on annual household income and working status. For pensioners, the level of co-payment can be 10% or 60% with different monthly maximum ceilings; for active workers, the co-payment level can be 40%, 50% or 60%, but no ceilings apply to these groups. There are also groups of people exempt from payment. Finally, most treatments for chronic conditions are subject to a 10% co-payment capped at 4,26€ per prescription [66–68].

### Study population

#### PC teams, GPs, and nurses

##### Recruitment and selection

The recruitment process follows both top-down and bottom-up approaches to identify and recruit PC teams and healthcare professionals.

A PC team is a group of GPs, nurses and other healthcare professionals who offer comprehensive care to a specific population. PC teams can work in one or more PC centres and large PC centres can accommodate more than one PC team. PC teams from all over Catalonia managed by the Catalan Health Institute will be assessed for eligibility (*n* = 287). A list of PC teams and their characteristics will be provided by the System for the Development of Research in Primary Care (SIDIAP). Pairs of PC teams will be randomly selected based on their location (rural/urban) and stratified according to certain non-initiation [9] predictors: PC teams located in urban areas will be stratified according to the number of practitioners in the PC team, the size of the catchment area population for each PC team, the socioeconomic status of the population and the proportion of immigrants; and PC teams located in rural areas will be stratified according to the socioeconomic status of the population. An ordered list of replacement PC teams with the same characteristics will be randomly generated for each pair of PC teams.

To avoid contamination between PC teams and community pharmacies, a maximum of one PC team will be selected for each municipality (in municipalities ≤ 100,000 inhabitants) or single PC teams per neighbourhood (in municipalities > 100,000 inhabitants). In the case of PC teams from adjacent municipalities, there must be a minimum distance of 3 km between each team’s working place. If a PC team does not fulfil the inclusion criteria, the following PC team from the list of replacements will be considered for participation. The inclusion of PC teams from all provinces will be ensured.

Randomly selected PC teams will be informed about the study and invited to participate. First, the PC Territorial Managers and team managers from the selected PC teams will be invited to explain and present them the project and will be asked to encourage GPs and nurses from their teams to take part in the study. If the team manager accepts to participate, the study will then be presented to the GPs and nurses in each team.

##### Inclusion criteria

Participating PC teams have to fulfil the following inclusion criteria: a) the PC team manager must be willing to participate in the study, commit to guaranteeing compliance with the ethical standards in the PC centre (see Ethics approval and consent to participate) and sign an informed consent for participation; and b) at least five GPs in urban areas or two GPs in rural areas who fulfil the inclusion criteria must be willing to participate at the moment of PC team inclusion. There is no minimum number of nurses required to participate.

To join the study, GPs and nurses have to fulfil the following inclusion criteria: a) to provide signed informed consent for participation in the clinical trial and the process evaluation; b) to attend the IMA intervention training entirely and c) not to anticipate a termination or interruption of employment (planning to change their place of work or taking sick/maternity/paternity leave) during the study period.

#### Community pharmacies and pharmacists.

##### Recruitment and selection

The recruitment process for community pharmacies will also follow top-down and bottom-up approaches to identify and recruit pharmacists. The research team will contact the General Council of Official Colleges of Pharmacists of Catalonia and the four Official Colleges of Pharmacists that exist in each of the four provinces of Catalonia to present the project. After the randomisation of the PC teams, each Official College of Pharmacists will be informed of the PC teams allocated to the intervention group; then, owners of community pharmacies that fulfil the inclusion criteria will be individually contacted and invited to participate in the study.

##### Inclusion criteria

Participating pharmacies have to fulfil the following criteria: a) it must be located within the area of the PC centres allocated to the intervention group, b) the pharmacy owner must be willing to participate and provide signed informed consent and c) if the pharmacy owner is not willing to participate, at least one other pharmacist who fulfils the inclusion criteria, must do so. To join the study, pharmacists have to a) sign an informed consent for participation in the clinical trial and the process evaluation, and b) attend the IMA intervention training.

#### Patients

##### Inclusion criteria

Patients will be identified from the electronic health records. All patients who a) are over 18 years old, b) receive a new prescription for lipid-lowering medication, antihypertensive medication, anti-platelet medication and/or antidiabetic medication (Table 1 shows the pharmacotherapeutic subgroups considered for study) from a participating GP during the seven-month study intervention period and c) do not refuse to participate in the study (see Ethics approval and consent to participate) will be included. A prescription is considered new if the patient has not had an active prescription from the same pharmacological group in the previous 6 months.

**Table 1:** Pharmacotherapeutic groups considered for the IMA intervention, following the ATC Classification System [69]

**A10 - Drugs used in diabetes**	A10A - Insulins and analogues	*A10AB* - *Insulins and analogues for injection, fast-acting*
		*A10AC - Insulins and analogues for injection, intermediate-acting*
		*A10AD - Insulins and analogues for injection, intermediate- or long-acting combined with fast-acting*
		*A10AE - Insulins and analogues for injection, long-acting*
	A10B - Blood glucose lowering drugs, excluding insulins	*A10BA - Biguanides*
		*A10BB - Sulfonylureas*
		*A10BD - Combinations of oral blood glucose-lowering drugs*
		*A10BF - Alpha-glucosidase inhibitors*
		*A10BG - Thiazolidinediones*
		*A10BH - Dipeptidyl peptidase 4 (DPP-4) inhibitors*
		*A10BJ - Glucagon-like peptide-1 (GLP-1) analogues*
		*A10BK - Sodium-glucose co-transporter 2 (SGLT2) inhibitors*
		*A10BX - Other blood glucose-lowering drugs, excl. Insulins*
**B01 - Antithrombotic agents**	B01A - Antithrombotic agents	*B01AC - Platelet aggregation inhibitors excl. Heparin*
**C02 - Antihypertensives**	C02A - Antiadrenergic agents, centrally acting	
	C02C - Antiadrenergic agents, peripherally acting	
	C02D - Arteriolar smooth muscle, agents acting on	
	C02K - Other Antihypertensives	
**C03 - Diuretics**	C03A - Low-ceiling diuretics, thiazides	
	C03B - Low-ceiling diuretics, excl. Thiazides	
	C03C - High-ceiling diuretics	
	C03D - Potassium-sparing agents	
	C03E - Diuretics and potassium-sparing agents in combination	
	C03X - Other diuretics	
**C07 - Beta blocking agents**	C07A - Beta blocking agents	
	C07B - Beta blocking agents and thiazides	
	C07C - Beta blocking agents and other diuretics	
	C07D - Beta blocking agents, thiazides and other diuretics	
	C07F - Beta blocking agents, other combinations	
**C08 - Calcium channel blockers**	C08C - Selective calcium channel blockers with mainly vascular effects	
	C08D - Selective calcium channel blockers with direct cardiac effects	
	C08G - Calcium channel blockers and diuretics	
**C09 - Agents acting on the renin-angiotensin system**	C09A - ACE inhibitors, plain	
	C09B - ACE inhibitors, combinations	
	C09C - Angiotensin II receptor blockers (ARBS), plain	
	C09D - Angiotensin II receptor blockers (ARBS), combinations	
	C09X - Other agents acting on the renin-angiotensin system	
**C10 - Lipid modifying agents**	C10A - Lipid modifying agents, plain	*C10AA - HMG coa reductase inhibitors*
		*C10AB - Fibrates*
		*C10AC - Bile acid sequestrants*
		*C10AD - Nicotinic acid and derivatives*
		*C10AX - Other lipid modifying agents*
	C10B - Lipid modifying agents, combinations	*C10BA - Combinations of various lipid modifying agents*
		*C10BX - Lipid modifying agents in combination with other drugs*

Each new prescription of the listed pharmacotherapeutic groups (Table 1) will be considered the index prescription. A patient can be included as many times as a new prescription from the groups under study is issued.

### Randomisation

Paired PC teams included in the study will be randomised (1:1) into two parallel groups using a computerised random number generator. Concealment of allocation was guaranteed at the PC team level: PC teams will not be randomised until both teams in each pair agree to participate in the study. However, at the patient level, it is not possible to guarantee concealment of allocation due to the intrinsic characteristics of the study design by clusters.

### Blinding

Due to the nature of the intervention, healthcare professionals and patients cannot be blind to it.

### Intervention

The IMA intervention aims to promote SDM between patients and healthcare professionals by providing the latter with the knowledge, skills and tools to increase patients’ health literacy and thus help the patient make an informed decision.

In Spain, the prescription process is not standardised and there is no guarantee that the patient will be involved in the decision-making process. When GPs consider that a patient is eligible for CVD or diabetes treatment, they usually explain the health problem and the prescribed treatment to the patient. Each GP decides how to provide this explanation. In other situations, GPs can recommend the treatment and it is the nurse who explains the healthcare problem and treatment to the patient during a follow-up consultation. As part of follow-up, nurses promote medication adherence and explore any potential side effects of the newly prescribed treatment. During the process of drug dispensing, community pharmacists are expected to explore patients’ knowledge and doubts about the medication, although this practice is not standardised either.

The foundations of the IMA intervention are SDM and the harmonisation and standardisation of clinical practice among healthcare professionals. GPs will be trained to use SDM during the time of consultation by informing the patient about their disease and the available treatment options with the help of decision aids (leaflets), and exploring their perspectives and queries before recommending a new pharmacological treatment, following the principles of the SDM model by Elwyn et al. [70, 71]. Finally, nurses and pharmacists will be encouraged to explore patients’ queries and use the decision aids to help standardise the discourse and improve collaboration among healthcare professionals.

As part of the implementation strategy of the IMA intervention, there are three inputs which are essential to achieve the intervention outcomes. First, top-down and bottom-up recruitment approaches are taken to increase professional engagement. Second, after the randomisation of the PC teams, healthcare professionals from the intervention group receive training on the IMA intervention, lasting 6 h. The training covers several aspects of non-initiation and other topics such as communication skills, health literacy and SDM. And lastly, decision aids have been designed to support the IMA intervention. These include one leaflet for each of the five pharmacotherapeutic groups and an *ad-hoc* website (available at: www.iniciadores.es). The leaflets will homogenise the intervention and provide tools to transmit the concepts of risk and benefit of the disease, treatment and alternatives. The leaflets contain a link to the website with a quick response code and this is considered a reliable source of information on CVD and diabetes. A full description of the intervention and its implementation strategy are described elsewhere [53].

Healthcare professionals in the control group will not receive training on SDM nor access to the decision aids and will be asked to provide usual care.

All participating professionals will receive a reinforcement session on the registry of clinical outcomes in the e-health records system and IMA-cRCT ethical standards.

**Fig. 2:**
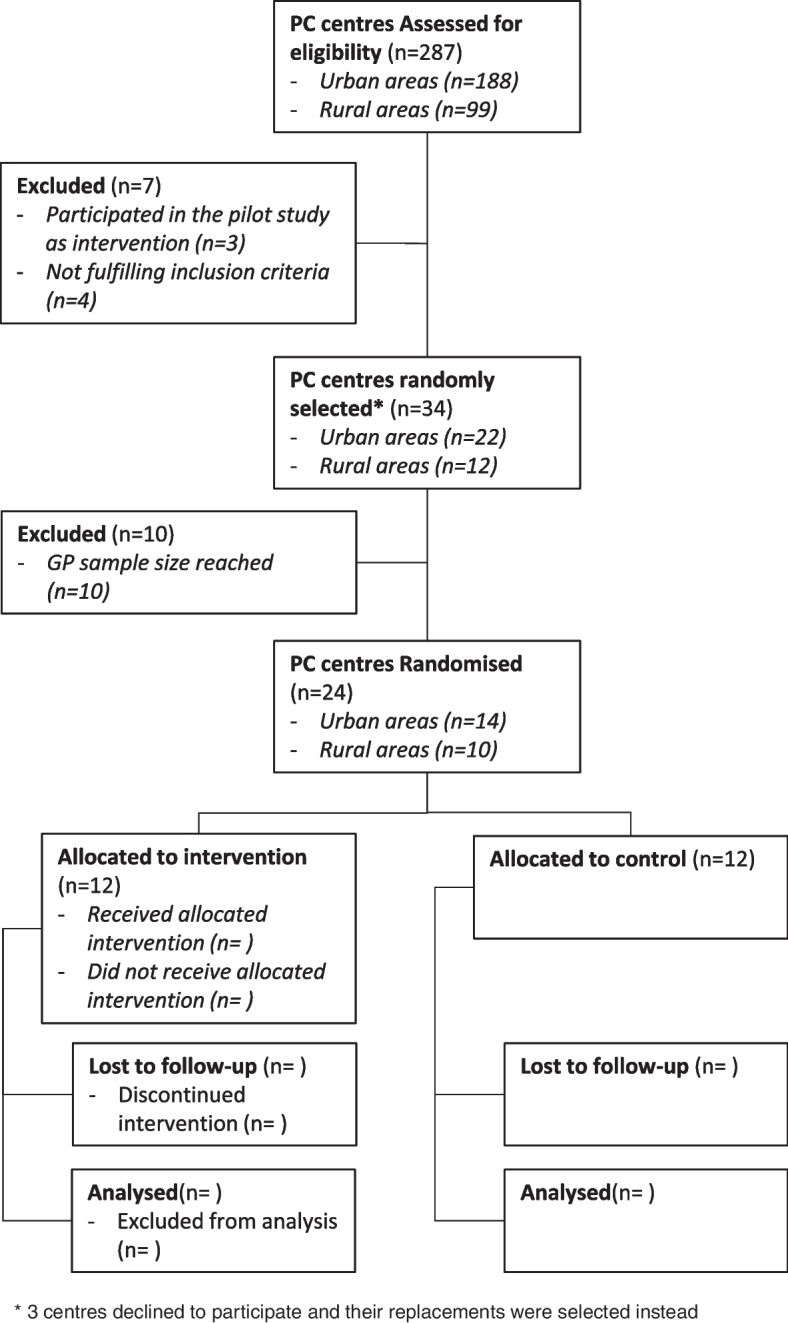
CONSORT flow diagram [72]

PC team pairs will be randomised to the intervention and control groups and training sessions will be scheduled. Upon completion of training, each pair of PC teams will start the 7-month intervention in March 2022. The study period will last 7 months to account for healthcare professionals’ summer holidays, which last up to a month. During this time, the intervention will be applied to each patient meeting inclusion criteria. Figure 2 shows the trial flow-chart based on CONSORT guidelines.

### Data collection

The SIDIAP database will be used to collect data for the evaluation of the effectiveness and cost-effectiveness of the IMA intervention. It implies the absence of any *ad-hoc* registry for the sake of the RCT.

SIDIAP gathers information from the electronic medical records of all patients seen by the public PC provider since 2010. This database provides information on patients’ sociodemographic and clinical data, including visits to primary care, health problems, sick leave periods, prescribed medicines, immunisations, laboratory results, clinical outcomes, and information on dispensed medication in any Catalan pharmacy [73]. All these records are dated.

The SIDIAP database is an encrypted, anonymised, secure database. It is managed by the Catalan Health Institute and CatSalut and provides real-world health data generated by the public health system in Catalonia to the scientific community under the legal and regulatory framework, following ethical principles, and maintaining transparency concerning the public program [74].

**Fig. 3:**
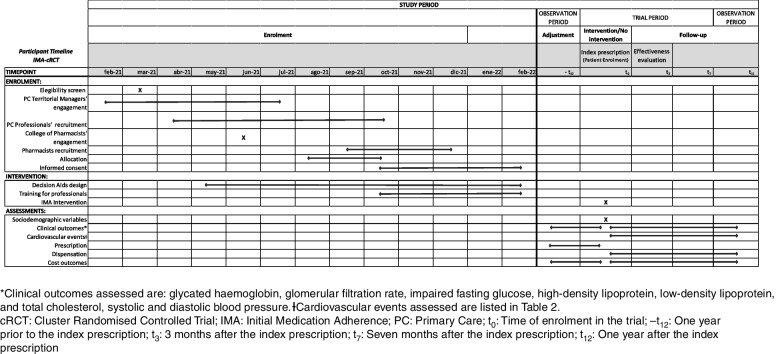
SPIRIT [55] figure

The SIDIAP database will be used to identify all patients that fulfil inclusion criteria to define the cohort of patients. They will be identified based on GP prescriptions. All variables will be collected for this cohort. All patient-related outcomes will be obtained from the encrypted and anonymised RWD databases. Patients’ personal information will not be provided to the research team. For each patient, data will be collected for the 12 months before the index prescription (for adjustment purposes) and from the subsequent 12 months (follow-up). Figure 3 depicts the observation periods in which the intervention is applied to patients. Information on healthcare professionals will be gathered through questionnaires at the training sessions [53].

**Table 2:** Events related to CVD and diabetes considered in measuring the effectiveness of the IMA intervention, as described in the 10th revision of the International Statistical Classification of Diseases and Related Health Problems (ICD-10) [69]

**E00-E90 - Endocrine, nutritional, and metabolic diseases**	E10-E14 - Diabetes mellitus	*E10 - Type 1 diabetes mellitus*
		*E11 - Type 2 diabetes mellitus*
		*E12 - Malnutrition-related diabetes mellitus*
		*E13 - Other specified diabetes mellitus*
		*E14 - Unspecified diabetes mellitus*
	E70-E90 - Metabolic disorders	*E78 - Disorders of lipoprotein metabolism and other lipidaemias*
**I00-I99 - Diseases of the circulatory system **	I10-I15 - Hypertensive diseases	*I10 - Essential (primary) hypertension*
		*I11 - Hypertensive heart disease*
		*I12 - Hypertensive renal disease*
		*I13 - Hypertensive heart and renal disease*
	I20-I25 - Ischaemic heart diseases	*I20 - Angina pectoris*
		*I21 - Acute myocardial infarction*
		*I22 - Subsequent myocardial infarction*
		*I23 - Certain current complications following acute myocardial infarction*
		*I24 - Other acute ischaemic heart diseases*
		*I24 - Chronic ischaemic heart disease*
	I30-I52 - Other forms of heart disease	*I50 - Heart failure*
		*I51 - Complications and ill-defined descriptions of heart disease*
	I60-I69 - Cerebrovascular diseases	*I60 - Subarachnoid haemorrhage*
		*I61 - Intracerebral haemorrhage*
		*I62 - Other nontraumatic intracranial haemorrhages*
		*I63 - Cerebral infarction*
		*I64 - Stroke, not specified as haemorrhage or infarction*
		*I65 - Occlusion and stenosis of precerebral arteries, not resulting in cerebral infarction*
		*I66 - Occlusion and stenosis of cerebral arteries, not resulting in cerebral infarction*
		*I67 - Other cerebrovascular diseases*
		*I68 - Cerebrovascular disorders in diseases classified elsewhere*
		*I69 - Sequelae of cerebrovascular disease*
	I70-I79 - Diseases of arteries, arterioles, and capillaries	*I70 - Atherosclerosis*
		*I71 - Aortic aneurysm and dissection*
		*I72 - Other aneurysm and dissection*
		*I73 - Other peripheral vascular diseases*
		*I74 - Arterial embolism and thrombosis*
		*I77 - Other disorders of arteries and arterioles*
		*I78 - Diseases of capillaries*
		*I79 - Disorders of arteries, arterioles and capillaries in diseases classified elsewhere*
**N00-N99 - Diseases of the genitourinary system**	N00-N08 - Glomerular diseases	*N06 - Isolated proteinuria with specified morphological lesion*
		*N08 - Glomerular disorders in diseases classified elsewhere*
	N17-N19 - Renal failure	*N17 - Acute renal failure*
		*N18 - Chronic kidney disease*
		*N19 - Unspecified kidney failure*

### Outcome measures

This study distinguishes between two different, but correlated, types of outcomes.

#### Effectiveness outcomes

##### Primary outcome measures

- *Initiation*: Patients who receive a new prescription will be considered initiators if they obtain their prescriptions in a community pharmacy during the following month [6]. Sensitivity analysis will be performed for a follow-up period of 3 months. Prescription and dispensation databases from SIDIAP will be compared to classify prescriptions as initiated and non-initiated.

##### Secondary outcome measures

- *Secondary adherence*: Implementation during the follow-up period will be calculated based on the proportion of days covered (PDC). PDC is the number of days in which 17 the prescribed medication is available divided by the number of days of the period when the prescription is active within the study period (365 days). This value ranges from 0 to 1 and is multiplied by 100 to obtain a percentage of adherence [75]. PDC has been proved to represent patient behaviour and treatment continuity [76, 77] accurately. Persistence will be defined as the time from initiation until discontinuation of the prescribed treatment, accepting a gap no longer than two months. Patients will be classified as adherent or otherwise by combining these two variables; that is, patients with PDC>80% during the follow-up year, with medication gaps up to 2 months, will be considered adherent.
- *Reduction of CVR*: The Framingham Risk Score will be calculated using clinical outcomes, like diabetes diagnosis, total cholesterol, high-density lipoprotein cholesterol, systolic and diastolic blood pressure, and sociodemographic variables including age and sex, and tobacco use [78] one year after the index prescription.

##### Other outcome measures

These data will be collected from the SIDIAP database for the 12 months after the index prescription.

- *Clinical parameters:* Clinical parameters assessed will depend on the diagnosis. Patients with type II diabetes: glycated haemoglobin, glomerular filtration rate, impaired fasting glucose; with dyslipidaemia: high-density lipoprotein, low-density lipoprotein and total cholesterol; and with hypertension: systolic and diastolic blood pressure.
- *Cardiovascular events:* Events related to CVD and diabetes, categorised according to the International Classification of Diseases, 10th version (ICD10). Table 2 shows the list of events considered.

#### Cost-effectiveness outcomes

All cost data will be collected from the SIDIAP database and will be understood to cover the use of healthcare and social resources and sick leave for each patient, 12 months before and after the index prescription.

The following direct costs will be considered: visits to PC (GP and nurse; on-site and home visits) and emergency room (PC or secondary care); referral to secondary care; hospital admissions (inpatient admissions and outpatient consultations); use of social care services (such as visits to the social worker); and outpatient diagnostic tests and medication use. Indirect costs considered include productivity losses (as sick leave).

#### Sociodemographic variables and diagnostics

Sociodemographic characteristics of the patients (sex, age, nationality, socioeconomic status, tobacco use and diagnoses at baseline), the prescribing GP (sex, age, nationality, years of work experience, specialisation and tutoring of medical residents) and the characteristics of the PC team (teaching centre, rurality, socioeconomic status of the reference area, number of GPs) will be gathered from the SIDIAP database. Additional information on all healthcare professionals (PC centre or pharmacy where they work, sex, occupation and years of work experience) will be also gathered through questionnaires [53].

### Sample size

According to previous results, the proportion of non-initiation of medications for CVD and diabetes in Catalonia is between 8–13% [18]. For sample size calculations, a proportion of 10% has been assumed. The sample size was estimated based on calculations for cluster randomised controlled trials [79]. Assuming a reduction in the incidence of non-initiation of 3%, a power of 80% and a significance level of 5%, given that the intracluster correlation coefficient for PC teams is 0.01, and assuming that, on average, each GP issues 30 new prescriptions of the selected medicines in 6 months, accounting for 10% of losses (due to incompleteness of data in clinical records), the necessary sample is 3,878 prescriptions and 130 GPs.

Considering 80% of urban PC centres in Catalonia and assuming a minimum number of five GPs per urban PC team and 2 per rural PC team, we will contact twenty-four PC teams to invite them to participate; fourteen from urban areas and ten from rural areas. PC teams will be included until the sample size is reached, i.e., 65 GPs are included in both the control and intervention groups.

### Statistical analysis

All analyses will be conducted following the intention to treat principle, including all patients treated by the GPs who fulfil inclusion criteria.

Poor registration of clinical outcomes and CVR data in electronic medical records may generate missing values. To deal with missing data, we will first explore the pattern of the missing data by using logistic regression models to test whether the observed variables predict the presence of missing data. If the existence of missing data is indeed explained by observed variables, a Missing at Random pattern will be assumed and multiple imputations with chained equations will be used to impute missing data. If possible, the imputation models will use any covariate that is predictive of missingness as well as all the variables that will be later used in the effectiveness and cost-effectiveness models [80]. The number of imputations will be determined by the fraction of missing information [81, 82]. The subsequent analyses will be conducted in each of the imputed datasets and the estimators will be pooled using Rubin’s rules [81].

A descriptive analysis, based on sociodemographic variables and health problems, will be performed to compare groups at baseline. Characteristics of participating healthcare professionals will also be compared between groups. Continuous variables will be presented with means and standard deviation; categorical variables will be presented with frequency and percentages. Differences between groups in these variables will be estimated using multilevel linear regression for continuous variables and multilevel logistic regression for categorical variables, considering the group as the independent variable in both cases.

The impact of the intervention will be assessed overall and for each pharmacotherapeutic subgroup, i.e., the 3rd level of the Anatomical Therapeutic Chemical (ATC) Classification System [83].

All models will be controlled for patient sociodemographic and clinical characteristics that have been described in the literature as predictors of non-initiation [9, 18] and which show statistically significant differences between intervention and control group at baseline; and will be performed using multilevel techniques. The basic unit of analysis will be either prescription or patient, based on the analysis. All analyses will be clustered at the level of PC team and GP.

#### Effectiveness

To assess the impact of the IMA intervention on initiation, a multilevel logistic regression will be estimated in which the dependent variable will be initiation and the independent variable will be the group.

A multilevel logistic regression model will be performed to compare the proportion of adherent patients between the intervention and control groups. In the model, the dependent variable will be adherence and the independent variable will be the group.

Multilevel repeated measure models will be used in which clinical parameters and CVR (the dependent variables) are considered several times, at diverse time points during the follow-up period.

The interaction ‘group x time’ will be used to evaluate the impact of the intervention (independent variables).

##### Sensitivity analysis

Per-protocol analyses will be performed including only patients who received a prescription by those GPs who attended both training sessions. Additionally, two complete case analyses will be performed: one considering only those patients attended by GPs who completed the 7-month study period, and another using only non-imputed data.

To assess uncertainty in the output of the models, a sensitivity analysis will be performed without controlling the models for baseline covariates to assess their effect on the results of the cRCT [84].

#### Economic evaluation

The economic evaluation of the IMA intervention will consist of two analyses: first, an economic analysis to assess the cost-effectiveness of the IMA-cRCT for the duration of the trial and, second, an economic model to extrapolate the results of the 12-month cRCT and estimate the long-term cost-effectiveness of the IMA intervention. The primary analysis, either for the trial or the model, will use an intention-to-treat approach; the key outcome will be the incremental cost-effectiveness ratio (ICER) of the IMA intervention compared to usual care.

The ISPOR guidelines on good research practices for cost-effectiveness analysis (CEA) alongside clinical trials [85] will be followed in the economic evaluation of the IMA intervention to improve its quality and therefore increase the value to decision-makers.

##### Short-term cost-effectiveness

The short-term CEA will consider 12-month individual patient-level clinical outcomes and costs from all participants included in the IMA-cRCT. The main CEA will be presented from a limited societal perspective [86].

##### Costs

For the analysis of the impact of the IMA intervention on total costs, costs from the limited societal perspective will be estimated by adding direct medical costs and indirect costs (i.e. productivity losses) [86].

Healthcare and social service use will be converted to monetary costs by multiplying each item by its tariffs, published in the Official Government Bulletin [87]. Medication cost paid by patients and by the Spanish National Health System is registered in the SIDIAP database. Sick leave will be used as a proxy for productivity losses, converted to monetary costs by using the minimum daily wage in Spain [88]. The price year used will be the most recent year for which official unit costs are available. Unit costs will be updated according to the 2023 Spanish Consumer Price Index (IPC). The cost of the IMA intervention will be calculated as part of the cost of study implementation, as described elsewhere [53], and used in a sensitivity analysis.

##### Health effects

The effect of the IMA intervention will be measured in terms of CVR, and improvements in medication initiation and secondary adherence.

##### Cost-effectiveness analysis

The difference in costs between groups will be estimated using multilevel generalised linear regression models with total costs as dependent variables. Due to the unpredictability in the distribution of costs, various distribution families and link functions will be tested and Akaike and Bayesian information criteria (AIC and BIC) will be used to choose the model with the best fit (usually the gamma distribution with a logistic link). The models will be controlled additionally for baseline costs (those incurred in the 12 months preceding the index prescription).

The difference in effects between groups will be estimated with multilevel regression models. For CVR reduction, multilevel linear regression models adjusted also for baseline CVR will be used. For secondary analyses, the difference in the probability of initiation and secondary adherence between groups will be estimated using a multilevel logistic regression with medication initiation or secondary adherence as the dependent variables.

The ICER will be calculated by dividing the difference in costs between groups by the difference in effects between groups.

##### Quantification of uncertainty

Sensitivity analyses will explore the robustness of the results. First, to evaluate the uncertainty surrounding the estimation of the ICER, one-way sensitivity analyses will explore the impact of 1) the perspective, by considering the health system perspective, accounting only for direct medical costs; 2) the unit cost of productivity losses by calculating loss of productivity as the average daily wage in Catalonia [89]; 3) the analytical approach (per-protocol and complete case analyses); and 4) the costs considered, by including the cost of implementing the IMA intervention. The bootstrapping method will then be used to assess uncertainty in the sampling distribution of the ICER by using a minimum of 5,000 bootstraps. Bootstrapped pairs of cost and effect differences will be plotted on cost-effectiveness planes.

#### Economic model

Economic models allow extrapolation of the cRCT’s results to evaluate the long-term cost-effectiveness of interventions assessed in short trials [90].

The model will be used to estimate long-term cost-effectiveness and cost-utility based on increased lifetime costs and effects for patients who receive the IMA intervention compared to those who receive usual care. The ICER of the IMA intervention in comparison to usual care will be reported in terms of cost per LYG, and the incremental cost-utility ratio (ICUR) in terms of cost per QALY gained from the IMA intervention.

The model will track patients included in the IMA-cRCT through CVR, any CVD-related events (Table 2) and death. It will contain estimates of average annual care costs and average utilities (quality of life) for each disease state, which will be accrued over 1-year cycle lengths until all patients enter the absorbing state of death (lifetime horizon, as is recommended for chronic conditions [91, 92]).

For the first year, information on transition probabilities will be obtained from the IMA-cRCT study. After the first year, information on transition probabilities will be obtained from RWD from the SIDIAP database and the existing literature. Yearly transitions will be incorporated into the model, which will consider adherence as a dynamic process. The economic model will be designed according to an ongoing epidemiological cohort study and based on previously published models [93, 94].

An annual discount rate of 3% for both costs and effects for the period of the main analysis beyond 12 months [95] will be applied.

##### Probabilistic sensitivity analysis (PSA)

PSA will be conducted using the Monte Carlo simulation method to assess parameter uncertainty. Each variable (event probability, costs or utilities) will be assigned the specific parameters of the associated distribution function [96], and values of the variables will be randomly sampled for each distribution. The model result will then be calculated according to the resampling values.

Probabilistic values of cost and effect differences will be plotted on cost-effectiveness planes. The willingness-to-pay (WTP) threshold for an additional QALY in Spain is set between 22,000–25,000€ [97]. The net monetary benefits of the IMA intervention compared to standard clinical practice will be calculated for different values of WTP per unit of outcome. Cost-effectiveness acceptability curves will be constructed showing the probability of the IMA intervention producing a net benefit for different values of WTP.

##### One way-sensitivity analysis

One-way sensitivity analysis will be conducted to evaluate methodological uncertainty. The parameters that show the greatest influence on the results, if possible, will be tested by one-way sensitivity analysis; variations on the perspective and costing will also be tested. For each one-way sensitivity analysis, a parameter of interest will be set to a specific value and the CEA and the PSA will be rerun to evaluate the robustness of the results regarding changes in this parameter.

The economic model will be constructed using Microsoft Excel and programmed in Visual Basic for Applications.

## Discussion

The IMA-cRCT is an ambitious research project: the burden associated with the intervention (training of healthcare professionals and development of the decision aids); the methods (recruitment of a large sample of healthcare professionals and patients, obstacles to accessing RWD, and the embedded process evaluation); and the dissemination of results (for implementation and scientific purposes) is high. However, the potential benefits to clinical practice, policy and research are notable.

The IMA intervention aims to standardise care, strengthen the interdisciplinary collaboration among healthcare professionals and promote patient empowerment. The implementation of the intervention aims to improve the quality of care for patients with CVD and diabetes.

Existing interventions to improve medication adherence are not theory-based, nor systematically developed or reported [29, 98]. The potential to produce effective, transferable interventions relies on the quality of the design, evaluation and dissemination processes. The IMA intervention will be, to the best of our knowledge, the first intervention to address initiation using a theory and evidence-based approach. It will also be the first study to evaluate the clinical and economic impact of these types of interventions since no studies have assessed the impact of the intervention on clinical outcomes and costs. Using the MRC Framework for the design of complex interventions [51, 52] to improve adherence is also an innovative approach; lessons learned will help with the design and assessment of further interventions.

Improving adherence is essential to achieving optimal clinical outcomes, which entails better control of the disease and thus a decrease in morbimortality and healthcare costs. The IMA intervention will implement a methodology to evaluate whether short-term investments to improve the care of patients can lead to savings in the long term, in both direct and indirect health costs.

Evidence on cost-effectiveness is also essential information for decision-making. Few studies evaluated the cost-effectiveness of interventions to improve adherence and even fewer used modelling techniques to assess their long-term cost-effectiveness [99]. This is partly explained by the difficulties in modelling adherence, which is a dynamic behaviour. Our study will provide information on the short and long-term cost-effectiveness of the IMA intervention. For the latter, we will build economic models, useful in analysing complex systems and accounting for dynamic behaviours [90]. The use of modelling techniques to extrapolate the results of an RCT to improve adherence is pioneering.

The IMA-cRCT is a pragmatic trial that uses RWD [100] to measure initiation, adherence and clinical outcomes. Using RWD in RCTs increases the transferability of results to real-life use. Pragmatic trials combine the scientific rigour of RCTs with the real-world nature of observational studies [101, 102]; its use is a singular approach that will improve the validity and generalisation of the results and their utility for end users [102].

RCTs are considered the gold standard for effectiveness evaluation [103, 104]. Translation to the clinical practice of interventions tested in RCTs is still a challenge. Implementation research aims to solve the science-to-service gap [49, 50]. The IMA-cRCT uses an effectiveness implementation hybrid design that evaluates the effects of the intervention while collecting information on implementation. This novel approach, together with dissemination to stakeholders and decision-makers, increases the chances of successful intervention implementation.

Improving adherence to medication and empowering patients to participate in the decision-making process and self-care is fundamental to the sustainability of the health system. However, the transferability of new interventions to clinical practice is challenging, especially when they are complex, behavioural interventions. Thus, the IMA intervention was designed in collaboration with stakeholders, taking into account theory generated using patients, healthcare professionals and knowledge on the context; a pilot study was conducted to assess the feasibility and acceptability of the intervention in real practice [54]; and evidence on short and long-term efficacy and cost-effectiveness will be provided to stakeholders, including decision-makers, health professionals and patient groups. Information on validity and generalisation of the assessment results will be provided to them, emphasising the pragmatism of the study design and the relevance of the study outcomes. This will include not only initiation and secondary adherence but clinical outcomes, reduction of CVR and projections of gains in life expectancy and QALYs. It is expected that the dissemination strategy will help achieve implementation.

### Strengths and limitations

Complex interventions, like the IMA intervention, contain several interacting components and present practical and methodological difficulties, such as standardising its design, adapting it to the local context and translating it into real practice [52]. We are aware that there may be many barriers that can hinder the implementation of an IMA intervention. The main ones are described below, together with the strategies for overcoming them.

Firstly, the workload of healthcare professionals is high, which could restrict their participation in the study and limit the fidelity of healthcare professionals to the intervention. To reduce the existing barriers to the participation of healthcare professionals, we involved healthcare professionals in the design of the IMA intervention and developed a brief and acceptable intervention tested in a pilot study [54], which is expected to increase fidelity to the intervention. Additionally, healthcare professionals from the intervention group will receive monthly newsletters with reminders and information related to the intervention. Simplified means of obtaining patients’ informed consent is also expected to facilitate the participation of GPs (see Ethics approval and consent to participate). Finally, all healthcare professionals will receive economic compensation for the time devoted to the training for the IMA intervention. Nevertheless, the top-down approach may limit the recruitment of healthcare professionals.

Periodic feedback will be provided throughout the study to remind healthcare professionals about the study and intervention. Since there will be no hard data to assess the fidelity of healthcare professionals to the intervention, they will be asked to self-report this during the process evaluation; per-protocol analyses under the perspective of the patients receiving the intervention will not be conducted.

RWD is used in this study to gather all data from patients and informed consent is obtained via simplified means (see Ethics approval and consent to participate). Since patients will not feel observed, bias is expected to be reduced (such as desirability bias and the Hawthorne effect) [105]. This also increases the pragmatism of the study. Even so, patients in the intervention group will receive the leaflets and be informed about the website, which can jeopardise the blinding of the intervention. Furthermore, since the IMA intervention is a one-shot intervention there exists the possibility that it will increase the risk of single dispensation of medication. To overcome it, both nurses and community pharmacists were invited to participate in the study to support and maintain GP’s intervention. The use of RWD to follow up patients for a year after the prescription will allow us to assess the impact of the IMA intervention on both non-initiation and single dispensing.

Using RWD allows the inclusion of a large sample in the study at an affordable cost and, consequently, this increases its power. Moreover, as a consequence of the large sample, external validity is improved as it increases the pragmatism of the study and the generalisation of results. Other studies have also demonstrated the validity of the SIDIAP database in epidemiological studies of CVD [106]. However, health registries are not designed for research purposes and some clinical information will likely be missing [73]. This limitation will not affect initiation or adherence outcomes that are based on hard data such as medication prescription and dispensing records but could affect the analysis based on clinical parameters and CVR. To minimise the effect of this limitation, power size calculations accounted for lost cases (due to incomplete data) and multiple imputations with chained equations will be used to deal with missing data.

The IMA intervention aims for coordination among healthcare professionals to improve adherence. Despite all GPs, nurses and pharmacists being invited to participate, not all of them accepted; consequently, patients will receive the intervention from their GPs, but perhaps not all will receive the intervention from participating nurses or pharmacists.

The ongoing COVID-19 pandemic can affect the execution of the IMA-cRCT. The training for healthcare professionals is intended to be imparted in person. However, if the restrictions do not allow big gatherings, it will be adapted to an online format. Likewise, the heavy workload as a consequence of the successive waves can affect the fidelity of healthcare professionals to the IMA intervention, not only due to lack of time but also to limitations on face-to-face consultations. These possible consequences will be assessed during the process evaluation, as well as their impact on the external validity of the study.

## Supplementary Information

**Additional file 1.**

**Additional file 2.**

## Data Availability

The research team is not the data owner as they are only re-using information that is the property of public health institutions. The data that support the findings of this study are available from SIDIAP but restrictions apply to the availability of these data, which were used under license for the current study, and so are not publicly available. Data are however available from the authors upon reasonable request and with permission of SIDIAP. Consequently, meta-data will not be published by the authors nor will data be identified with a DOI. The tools designed for the IMA intervention are publically available at www.iniciadores.es.

## Abbreviations

COVID-19: Coronavirus disease
CEA: Cost-effectiveness analysis
cRCT: Cluster randomised controlled clinical trial
CVD: Cardiovascular disease
CVR: Cardiovascular risk
GP: General practitioner
ICER: Incremental cost-effectiveness ratio
IMA: Initial medication adherence
LYG: Life years gained
MRC: Medical research council
PC: Primary care
QALY: Quality-adjusted life years
RCT: Randomised controlled trial
RWD: Real-world data
SDM: Shared decision-making
