# Correction: Effectiveness and cost‑effectiveness of an intervention to improve Initial Medication Adherence to treatments for cardiovascular diseases and diabetes in primary care: study protocol for a pragmatic cluster randomised controlled trial and economic model (the IMA‑cRCT study)

**DOI:** 10.1186/s12875-024-02694-w

**Published:** 2024-12-27

**Authors:** Alba Sanchez‑Vinas, Carmen Corral‑Partearroyo, Montserrat Gil‑Girbau, M. Teresa Penarrubia‑Maria, Carmen Gallardo‑Gonzalez, Maria‑del‑Carmen Olmos‑Palenzuela, Ignacio Aznar‑Lou, Antoni Serrano‑Blanco, Maria Rubio‑Valera

**Affiliations:** 1 Health Technology Assessment in Primary Care and Mental Health (PRISMA) Research Group, Institut de Recerca Sant Joan de Deu, Santa Rosa 39‑57, Esplugues de Llobregat, 08950 Spain; 2 Centro de Investigacion Biomedica en Red de Epidemiologia y Salud Publica (CIBERESP), Madrid, Spain; 3 Facultat de Medicina I Ciencies de La Salut, Universitat de Barcelona, c. Casanova 143, Barcelona, 08036 Spain; 4 Department of Paediatrics, Obstetrics, Gynaecology and Preventive Medicine, Univ Autonoma de Barcelona, Bellaterra, Spain; 5 Parc Sanitari Sant Joan de Deu, Doctor Antoni Pujadas 42, Sant Boi de Llobregat, 08830 Spain; 6 Centre d’Atencio Primaria Bartomeu Fabres Anglada, Direccio D’Atencio Primaria Regio Metropolitana SudInstitut Catala de La Salut, Barcelona, Spain; 7 Unitat de Suport a La Recerca Regio Metropolitana SudFundacio Institut Universitari Per a La Recerca a L’Atencio Primaria de Salut Jordi Gol I Gurina (IDIAPJGol), Barcelona, Spain

**Correction: BMC Prim Care 23, 170 (2022)**

10.1186/s12875-022-01727-6

Following publication of the original article [1], the authors reported errors in the ‘Method’ section and ‘Ethics approval and consent to participate’. Corrected data were highlighted in bold in the below table.

**Table:** 

Incorrect	Correct
**Methods**	
the necessary sample size was 3,878 **patients** and 130 GPs	the necessary sample is 3,878 **prescriptions** and 130 GPs
from a participating GP during the **six-month** study intervention period	from a participating GP during the **seven-month** study intervention period
Upon completion of training, each pair of PC teams will start the 7-month intervention in March** 2021**	Upon completion of training, each pair of PC teams will start the 7-month intervention in March** 2022**
**Ethics Approval and Consent to Participate**	
Simplified informed consent requires that the same information stated under the **Article 29(2)** of the Regulation (EU) No 536/2014	Simplified informed consent requires that the same information stated under the **Article 30** of the Regulation (EU) No 536/2014

The original article [1] has been corrected.
