# Supplementary material for: Effectiveness and cost-effectiveness of an intervention to improve Initial Medication Adherence to treatments for cardiovascular diseases and diabetes in primary care: study protocol for a pragmatic cluster randomised controlled trial and economic model (the IMA-cRCT study)
Source: BMC Prim Care. 2022 Jul 5;23:170. doi: 10.1186/s12875-022-01727-6 (PMC9255541; doi:10.1186/s12875-022-01727-6)
Supplement: Supplementary file 2 — Additional file 2. [file 12875_2022_1727_MOESM2_ESM.pdf]

Effectiveness and cost-effectiveness of the Initial Medication Adherence intervention: a cluster randomised control trial, economic model and process evaluation (IMA-cRCT)

PRECIS-2 Table of scores for trial domains

	Domain	*Score*	*Rationale*
**1**	Eligibility Criteria	5	Participants receiving the intervention are identical to those in usual care. All patients >18 years old who are treated by a participating PC physician that receive a new prescription for CVD or diabetes and do not refuse to participate in the study will be included in the study.
**2**	Recruitment Path	5	Recruitment takes place in Primary Care Centres (PCC) during a regular patient visit when a new treatment for cardiovascular disease or diabetes is prescribed. Informed consent will be obtained by simplified means. Therefore, all patients who fulfil the inclusion criteria and do not refuse to participate will be included in the study. Potential trial participants visit the PCC on their behalf, without any special incentive.
**3**	Setting	5	Identical settings to usual care. The study will be developed in the Spanish PC setting. The participant PC centres will be randomly selected from PC centres all over Catalonia (Spain).
**4**	Organisation of Intervention	4	General practitioners, community pharmacists and nurse practitioners will receive training on the intervention basis and tools and how to use them. The training will cover the motives of non-initiation, communication skills and shared-decision making. The healthcare professionals will also be trained to use technical support tools, such as leaflets, a website and a prescription alarm to remember to apply the IMA intervention. The professionals in the intervention group will have access to decision tools and an e-prescription alarm that are not available in usual care. Apart from that, the intervention will be applied in an identical setting to usual care, with identical resources, provider expertise and organisation of care delivery.
**5**	Flexibility of experimental intervention - delivery	4	The study intervention aims to provide healthcare professionals with the knowledge, skills and tools to help the patient make an informed decision. A brief and flexible intervention is proposed to facilitate the scalability and transferability of the intervention. The intervention will provide healthcare professionals with specific training (6h) and technical support tools (informative leaflets, website and prescription alarm) to try to harmonize and standardize the usual care of PC professionals when prescribing a new medication. The methodology of how to deliver the intervention is not rigidly prescriptive in the protocol, although it states how to administer the intervention and there are measures in place to monitor the compliance of the professionals with the protocol. Monitoring of the intervention will be conducted to assess and improve compliance with the intervention.
**6**	Flexibility of experimental intervention - adherence	5	Health professionals will be monitored to identify barriers to the implementation of the intervention and encouraged to adhere to it. However, adherence to the intervention will not be used as exclusion criteria and no specific measures to

			improve adherence have been proposed although reminders will be sent to study participants to encourage the use of the intervention.
**7**	Follow up	5	All the information to assess the effectiveness and cost-effectiveness of the intervention will be collected from routine records that are registered in the electronic clinical history. Patients will be followed according to current clinical guidelines applied at the PC centres and the follow-up will be the same for the intervention and usual care patients.
**8**	Outcome	2	The primary outcome is the improvement of initial medication adherence in 3 percentual points. Adherence problems are associated with poorer health outcomes and long-term health complications of cardiovascular disease (CVD) and diabetes. However, this is a surrogate outcome. Non-initiation is associated with higher costs, mainly generated by productivity losses and increased number of home visits, which suggest worse disease progress. On the other hand, CVD and diabetes are highly prevalent diseases that cause high mortality, disability and economic burden. Despite the incidence of non-initiation in the prevention and treatment of CVD and diabetes is lower than in other diseases, studies show that non-adherence to treatments for CVD and diabetes worsens control of the disease and clinical results, increasing morbidity and mortality and direct and indirect health costs. Overall, the outcome is of the utmost relevance for stakeholders and decision-makers, but the primary outcome is not a priority for the patients.
**9**	Analysis	5	A very pragmatic approach using intention to treat with all available data. Real-world data from electronic health records will be used in the analysis.

PRECIS-2 evaluation of the IMA-cRCT
Available online: https://www.precis-2.org/Trials/Details/679

PRECIS-2 wheel scheme of the IMA-cRCT

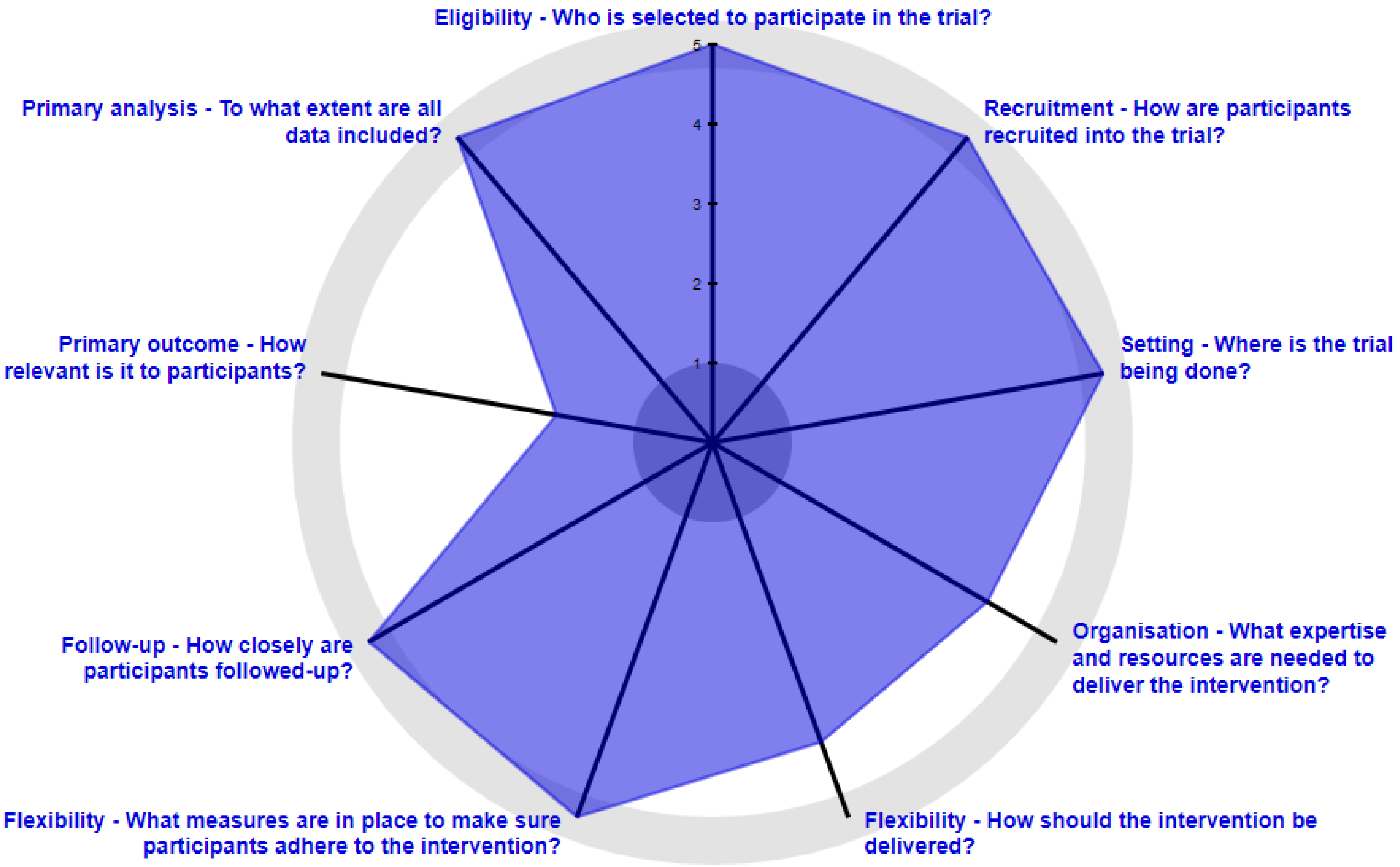

PRECIS-2 evaluation of the IMA-cRCT
Available online: https://www.precis-2.org/Trials/Details/679
